# Decrease in Rate of Opioid Analgesic Overdose Deaths — Staten Island, New York City, 2011–2013

**Published:** 2015-05-15

**Authors:** Denise Paone, Ellenie Tuazon, Jessica Kattan, Michelle L. Nolan, Daniella Bradley O’Brien, Deborah Dowell, Thomas A. Farley, Hillary V. Kunins

**Affiliations:** 1 Bureau of Alcohol, Drug Use, Prevention, Care and Treatment, New York City Department of Health and Mental Hygiene; 2 Office of Noncommunicable Diseases, Injury and Environmental Health, National Center for Injury Prevention and Control, CDC; 3 The Public Good Projects, New York City

From 2000 to 2011, the rate of unintentional drug poisoning (overdose) deaths involving opioid analgesics increased 435% in Staten Island, from 2.0 to 10.7 per 100,000 residents. During 2005–2011, disparities widened between Staten Island and the other four New York City (NYC) boroughs (Bronx, Brooklyn, Manhattan, and Queens) (1); in 2011, the rate in Staten Island was 3.0–4.5 times higher than in the other boroughs. In response, the NYC Department of Health and Mental Hygiene (DOHMH) implemented a comprehensive five-part public health strategy, with both citywide and Staten Island–targeted efforts: 1) citywide opioid prescribing guidelines, 2) a data brief for local media highlighting Staten Island mortality and prescribing data, 3) Staten Island town hall meetings convened by the NYC commissioner of health and meetings with Staten Island stakeholders, 4) a Staten Island campaign to promote prescribing guidelines, and 5) citywide airing of public service announcements with additional airing in Staten Island. Concurrently, the New York state legislature enacted the Internet System for Tracking Over-Prescribing (I-STOP), a law requiring prescribers to review the state prescription monitoring system before prescribing controlled substances. This report describes a 29% decline in the opioid analgesic–involved overdose death rate in Staten Island from 2011 to 2013, while the rate did not change in the other four NYC boroughs, and compares opioid analgesic prescribing data for Staten Island with data for the other boroughs. Targeted public health interventions might be effective in lowering opioid analgesic–involved overdose mortality rates.

In NYC, the rate of opioid analgesic–involved overdose deaths increased 57% from 2005 to 2011, from 2.1 to 3.3 per 100,000 residents. While rates increased citywide, the rate in Staten Island increased 257% during the same period, from 3.0 to 10.7 per 100,000 residents (Figure). In April 2011, DOHMH reported citywide opioid analgesic–involved overdose mortality, highlighting the disproportionately high rates in Staten Island (2). This report received substantial media coverage, particularly among Staten Island local news outlets. In November 2011, DOHMH published opioid prescribing guidelines for general medical providers with the following key messages: 1) a 3-day supply of short-acting opioid analgesic is usually sufficient for acute pain, 2) avoid prescribing opioid analgesics for chronic noncancer pain, 3) avoid high-dose opioid analgesic prescriptions, and 4) avoid prescribing opioid analgesics to patients taking benzodiazepines (3). In January 2013, DOHMH released opioid prescribing guidelines for emergency departments (4) that were adopted citywide by 39 emergency departments, including both of Staten Island’s hospitals.

Throughout 2013, DOHMH met in Staten Island with local hospital, addiction treatment, and syringe exchange programs, as well as local politicians to share overdose mortality trends and guidelines. In June 2013, the commissioner of health held two conferences for Staten Island physicians on judicious opioid prescribing. These guidelines were promoted to Staten Island prescribers via one-to-one office educational visits in which DOHMH recommendations, resources, and tools were disseminated. During 2012–2014, DOHMH aired two television advertisements highlighting the risks of opioid analgesics citywide, with additional airtime in Staten Island. These interventions occurred in close temporal proximity to the enactment and media coverage of I-STOP, state legislation implemented in August 2013 that requires providers to consult the state Prescription Monitoring Program, a registry of controlled-substance prescriptions filled by New Yorkers, before prescribing or dispensing Schedule II, III, or IV controlled substances.

To evaluate the impact of the public health interventions, DOHMH assessed changes in unintentional opioid analgesic–involved overdose mortality rates and changes in opioid analgesic prescribing patterns. Mortality data were derived from two linked sources, NYC death certificates and toxicology findings from the Office of the Chief Medical Examiner. Deaths were defined as unintentional drug poisoning (overdose) if the medical examiner determined manner of death as accidental and the underlying or multiple cause code was assigned an ICD-10 code of X40–X44, F11–F16, or F18–F19 (excluding F-codes with 0.2 or 0.6 third digit). Toxicology metabolites were abstracted from medical examiner files and linked to death certificate data.

Toxicology findings were used to describe the drugs involved in overdose deaths. Methadone-involved overdose deaths were reported separately, because there are approximately 30,000 New Yorkers maintained on methadone for opioid use disorders. Staten Island opioid analgesic–involved overdose rates were compared with the other four NYC boroughs combined. Overall, overdose rates also were assessed to determine whether changes in opioid analgesic–involved overdose rates were offset by changes in other drug poisonings, principally heroin.

Data for opioid analgesic prescriptions filled by NYC residents were derived from the New York State Prescription Monitoring Program. DOHMH assessed median day supply and the fill rates of prescriptions and high morphine equivalent–dose prescriptions (>100 morphine milligram equivalents) (5) by borough of patient residence.

Age-adjusted rates were calculated using NYC population estimates for the period 2000–2013 and the U.S. Census 2000 standard population. To evaluate the impact of the public health interventions, prescription rates were compared annually and for the fourth quarters (October–December) during 2011–2013. Given that both office educational visits with Staten Island prescribers and implementation of I-STOP occurred in the third quarter of 2013, the fourth quarter of 2013 was compared with the fourth quarters of 2011 and 2012. Rate changes were tested using z-tests and 95% confidence intervals; comparisons were based on gamma confidence intervals distribution (6).

From 2000 to 2011, Staten Island residents had the highest rate of opioid analgesic–involved overdose mortality in NYC. From 2005 to 2011, the rate increased 257% in Staten Island, compared with a 44% increase in the other four boroughs combined. After implementation of the public health initiatives, opioid analgesic mortality rates decreased 29% from 2011 to 2013, from 10.7 to 7.6 per 100,000 Staten Island residents (Table 1). In comparison, the rate for the other four boroughs combined did not change from 2011 to 2013 (2.6 per 100,000 residents, for both years). Among Staten Island residents, the rate of heroin-involved overdose deaths fluctuated but had a net increase of 39% from 2011 to 2013, from 6.2 in 2011 to 8.6 per 100,000 residents in 2013. Among the other four boroughs combined, heroin-involved overdose deaths increased 35% during the same period (from 3.7 in 2011 to 5.0 per 100,000 residents in 2013). In Staten Island, overall drug-involved overdose deaths decreased 4% from 2011 to 2013, from a rate of 18.4 to 17.6 per 100,000 residents. During that period, the rate for the other four boroughs increased 20%, from 7.9 to 9.5 per 100,000.

The median day supply for filled opioid analgesic prescriptions for Staten Island residents was unchanged during 2011–2013 (30 days). In contrast, the median day supply for the other four boroughs was lower, but increased from 2011 to 2013, from 15 to 20 days (Table 2).

In 2011, Staten Island residents filled opioid analgesic prescriptions at a higher rate (502.0 per 1,000 residents) than did residents of the other four boroughs (236.7) and filled high-dose prescriptions at rates three times higher (132.4) than residents of the other boroughs (40.7) (Table 2). In 2012, the rate of opioid analgesic prescriptions filled decreased in all boroughs, whereas rates of high-dose prescriptions increased slightly. Compared with 2011, in 2013 the opioid analgesic prescriptions fill rate continued to decrease for residents of all boroughs, by 9.8% in Staten Island (to 452.9 per 1,000 residents) and by 8.2% (to 217.2) elsewhere. The rate of high dose prescriptions decreased 8.2% (to 121.6 per 1,000 residents) in Staten Island while increasing 4.7% (to 42.6) in the other four boroughs. The decrease in Staten Island rates of high dose prescriptions continued in the final quarter of 2013.

## Discussion

After implementation of targeted and general public health initiatives, Staten Island saw 2 years of decreases in opioid analgesic high-dose prescribing and opioid analgesic–involved overdose mortality; the decreases followed 11 years of increases. In contrast, high-dose prescribing in the other four NYC boroughs increased without changes in opioid analgesic–involved overdose mortality rates. In addition, the decreases in opioid analgesic overdoses on Staten Island were not offset by increases in heroin-involved overdose mortality.

Decreases in opioid analgesic–involved overdose mortality have been reported from Wilkes County, North Carolina (7), Utah (8), Washington (9), and Florida (10). Each county or state employed a tailored strategy or combination of strategies to address opioid analgesic–involved overdose deaths, most of which included policy and clinical interventions. NYC employed both a general and geographically targeted approach, similar to Wilkes County, aiming to reach the entire NYC population and all prescribers, but found decreased mortality only in the targeted Staten Island area that received the most intensive interventions.

The findings in this report are subject to at least three limitations. First, although decreases were observed in both high-dose prescribing and opioid analgesic–involved mortality rates, it is not known whether decedents had taken prescribed or nonprescribed opioids, nor at what doses. Both decreases might be attributed to decreased risk for persons prescribed opioids or a decrease in the amount of opioids available for diversion to nonprescribed use. Second, law enforcement efforts to decrease the supply of diverted opioids or to reduce malpractice were not considered, although these efforts occurred during the period of the public health interventions. Finally, although the public health interventions were followed by a reduction in opioid analgesic–involved overdose mortality rates in Staten Island, it is not possible to determine the extent of each intervention’s contribution to the decline.

Despite limitations, the fact that some of the initiatives were statewide or citywide (I-STOP, prescribing guidelines, and public service announcements), whereas others were Staten Island–specific (local media, local community engagement and conferences, tailored advertising messages, and office educational visits with prescribers) suggests that the community-specific initiatives might have been key to the decreases in Staten Island without corresponding decreases citywide. Staten Island’s size (500,000 pop.) and relative geographic separation from the other four NYC boroughs also might have enhanced its saturation with prevention messages and strategies. This tailored and intensive approach might be effective in other jurisdictions with high rates of opioid analgesic–involved mortality.

What is known already?

Opioid analgesic–involved overdose mortality is a serious public health issue. In New York City, the rate of opioid analgesic–involved overdose deaths increased 57% citywide, from 2005 to 2011. However, in one borough, Staten Island, the rate increased 257% during that period.

What is added by this report?

This report shows that data-driven, multi-pronged public health strategies, including judicious prescribing guidelines, office educational visits with providers, dissemination of timely data reports, and media campaigns, might contribute to a reduction in the rate of opioid analgesic–involved overdose deaths in Staten Island.

What are the implications for public health practice?

Targeted public health interventions appear effective in lowering opioid analgesic–involved overdose mortality rates; the interventions in Staten Island might be replicated by other health departments.

## Figures and Tables

**FIGURE:**
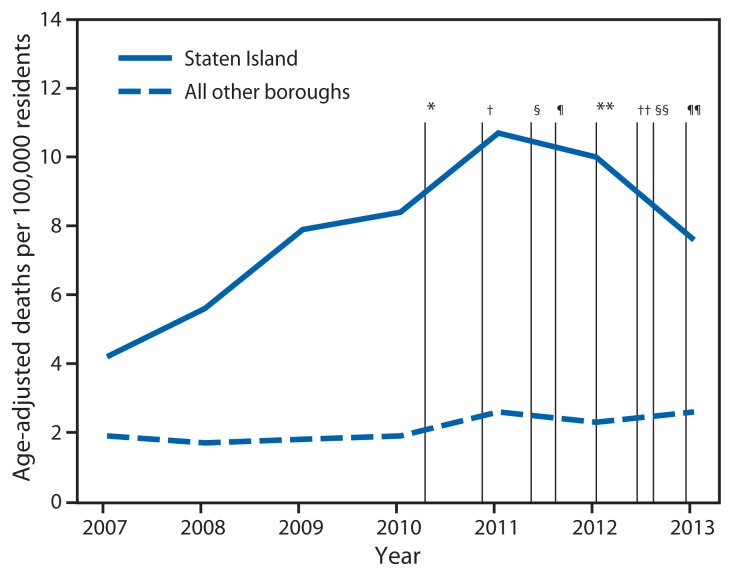
Age-adjusted rate of unintentional drug poisoning (overdose) deaths involving opioid analgesics, by borough of residence, and New York City public health interventions — 2007–2013 **Source:** New York City Office of the Chief Medical Examiner and New York City Department of Health and Mental Hygiene 2007–2013. * April 2011: Distributed a data brief citywide that highlighted overdose mortality and prescription use in Staten Island. ^†^ November 2011: Distributed opioid prescribing guidelines to all providers citywide. ^§^ May 2012: Ran first public service announcement campaign citywide. ^¶^ August 2012: State legislation passed mandating use of the prescription monitoring program. ** January 2013: Distributed opioid prescribing guidelines to emergency departments citywide. ^††^ June 2013: Town halls convened in Staten Island by New York City commissioner of health and meeting held with Staten Island stakeholders. Implemented detailing campaign to promote opioid prescribing guidelines to prescribers in Staten Island. ^§§^ August 2013: Statewide mandatory prescriber use of prescription monitoring program begun. ^¶¶^ December 2013: Ran second public service announcement campaign citywide with additional targeted airing in Staten Island.

**TABLE 1:** Number and rate per 100,000 residents* of unintentional drug poisoning (overdose) deaths involving any drug, heroin, or opioid analgesics,† by borough of residence§ — New York City, 2011–2013

Borough of residence	2011		2012		2013		% rate change from 2011 to 2013
		
	Total	(Rate)	Total	(Rate)	Total	(Rate)	
**New York City**							
Any drug	**567**	(8.5)	**660**	(9.8)	**672**	(9.9)	+16.4¶
Heroin	**253**	(3.8)	**339**	(5.0)	**352**	(5.2)	+36.8¶
Opioid analgesics	**201**	(3.0)	**181**	(2.7)	**197**	(2.9)	−3.3
**Staten Island**							
Any drug	**69**	(18.4)	**74**	(19.9)	**64**	(17.6)	−4.3¶
Heroin	**22**	(6.2)	**36**	(10.1)	**32**	(8.6)	+38.7¶
Opioid analgesics	**40**	(10.7)	**37**	(10.0)	**28**	(7.6)	−29.0¶
**Other four boroughs**							
Any drugs	**498**	(7.9)	**586**	(9.3)	**608**	(9.5)	+20.3¶
Heroin	**231**	(3.7)	**303**	(4.8)	**320**	(5.0)	+35.1¶
Opioid analgesics	**161**	(2.6)	**144**	(2.3)	**169**	(2.6)	0.0

**Source:** Office of Chief Medical Examiner, New York City.

* Age-adjusted rates are calculated using intercensal estimates updated in December 2014, and are weighted to U.S. Census Standard 2000.

† The drug types are not mutually exclusive; most overdoses involved more than one substance.

§ Analysis limited to residents of Staten Island and the other four New York City boroughs (Bronx, Brooklyn, Manhattan, and Queens), based on data reported on death certificates.

¶ Statistically significant rate change (p<0.05), determined by z-tests and 95% confidence interval comparisons based on gamma confidence intervals distribution.

**TABLE 2:** Number and rate per 1,000 residents* of annual and quarterly (October–December) opioid analgesic prescriptions and high morphine equivalent dose prescriptions received,† by borough of residence§ — New York City, 2011–2013

Borough of residence	2011		2012		2013		% rate change from 2011 to 2013¶
		
	Total	(Rate)	Total	(Rate)	Total	(Rate)	
**New York City**							
Opioid analgesic prescriptions	**2,172,238**	(251.9)	**2,167,719**	(248.4)	**2,029,541**	(230.6)	−8.5%
High morphine equivalent dose prescriptions	**395,605**	(45.8)	**419,476**	(48.1)	**413,801**	(47.0)	+2.6%
**Staten Island**							
Opioid analgesic prescriptions	**251,705**	(502.0)	**245,449**	(487.3)	**231,139**	(452.9)	−9.8%
High morphine equivalent dose prescriptions	**65,310**	(132.4)	**66,007**	(133.7)	**60,866**	(121.6)	−8.2%
**Other four boroughs**							
Opioid analgesic prescriptions	**1,920,544**	(236.7)	**1,922,270**	(234.1)	**1,798,402**	(217.2)	−8.2%
High morphine equivalent dose prescriptions	**330,276**	(40.7)	**353,469**	(43.1)	**352,935**	(42.6)	+4.7%
**Median days supply of drug**							
New York City	**16**	—	**20**	—	**20**	—	—
Staten Island	**30**	—	**30**	—	**30**	—	—
Other four boroughs	**15**	—	**17**	—	**20**	—	—
**Borough of residence**	**October–December 2011**		**October–December 2012**		**October–December 2013**		**% rate change from October–December 2011 to October–December 2013** ¶
		
	**Total**	**(Rate)**	**Total**	**(Rate)**	**Total**	**(Rate)**	

**New York City**							
Opioid analgesic prescriptions	**553,650**	(64.2)	**531,109**	(60.9)	**496,100**	(56.3)	−12.3%
High morphine equivalent dose prescriptions	**107,013**	(12.4)	**105,477**	(12.1)	**104,886**	(11.9)	−4.0%
**Staten Island**							
Opioid analgesic prescriptions	**63,676**	(127.0)	**58,234**	(115.3)	**56,769**	(110.7)	−12.8%
High morphine equivalent dose prescriptions	**17,098**	(34.7)	**15,611**	(31.5)	**15,011**	(29.9)	−13.8%
**Other four boroughs**							
Opioid analgesic prescriptions	**489,974**	(60.4)	**472,875**	(57.6)	**439,331**	(53.0)	−12.3%
High morphine equivalent dose prescriptions	**89,915**	(11.1)	**89,866**	(11.0)	**89,875**	(10.9)	−1.8%

**Source:** Bureau of Narcotic Enforcement, Prescription Drug Monitoring Program, New York State Department of Health, 2011–2013.

* Age-adjusted rates are calculated using intercensal estimates updated in December 2014, and are weighted to U.S. Census Standard 2000.

† Analysis includes prescriptions written for Schedule II (excluding codeine-2) and hydrocodone. Prescriptions written by veterinarians, or written under institutional licenses, or prescriptions with missing prescriber ID, or missing patient ID are excluded. Morphine equivalent dose (MED) is the equivalent of 1 mg of morphine; high MED prescriptions are greater than 100 MED.

§ Analysis limited to residents of Staten Island and the other four New York City boroughs (Bronx, Brooklyn, Manhattan, and Queens).

¶ All rate changes were statistically significant (p<0.05).
